# The causal association between COVID-19 and herpes simplex virus: a Mendelian randomization study

**DOI:** 10.3389/fimmu.2023.1281292

**Published:** 2023-12-11

**Authors:** Ming Yan, Li-yuan Xiao, Martin Gosau, Reinhard E. Friedrich, Ralf Smeets, Ling-ling Fu, Hong-chao Feng, Simon Burg

**Affiliations:** ^1^ Department of Oral and Maxillofacial Surgery, Guiyang Hospital of Stomatology, Guiyang, China; ^2^ Department of Oral and Maxillofacial Surgery, University Medical Center Hamburg-Eppendorf, Hamburg, Germany; ^3^ Department of Oral and Maxillofacial Surgery, Division of Regenerative Orofacial Medicine, University Medical Center Hamburg-Eppendorf, Hamburg, Germany

**Keywords:** COVID-19, herpes simplex virus, Mendelian randomization, causal effect, association

## Abstract

**Introduction:**

The coronavirus disease 2019 (COVID-19) has emerged as a main global public health challenge. Additionally, herpes simplex virus type-1 (HSV-1) and type 2 (HSV-2) are widespread viruses that can cause orolabial herpes and genital herpes. Several clinical case reports have declared a possible association between the two, however, the causal relationship between them has not been clarified.

**Methods:**

This study utilized a Mendelian randomization (MR) approach for causality assessment between COVID-19 infection and HSV infection based on the latest public health data and Genome-Wide Association Study (GWAS) data. Multiple causal estimation methods, such as IVW, weighted median, simple mode, and weighted mode, were employed to validate the causal relation between COVID-19 infection and HSV infection, with COVID-19 infection, COVID-19 hospitalization, and severe COVID-19 as exposures, and HSV1/2 infection as the outcome. A reverse MR analysis was subsequently performed.

**Results:**

MR analysis exhibited that COVID-19 infection was relevant to a reduced risk of HSV1 infection (p=7.603239e−152, OR=0.5690, 95%CI=0.5455−0.5935, IVW). Regarding the effect of COVID-19 infection on HSV2, MR analysis suggested that COVID-19 infection was correlated with an augmented risk of HSV2 infection (p=6.46735e−11, OR=1.1137, 95%CI=1.0782−1.1502, IVW). The reverse MR analysis did not demonstrate a reverse causal relationship between HSV and COVID-19.

**Discussion:**

Altogether, COVID-19 infection might cause a decreased risk of HSV1 infection and an elevated risk of HSV2 infection.

## 1 Introduction

Coronavirus disease 2019 (COVID-19) has emerged as a major public health challenge worldwide, leading to infections affecting nearly the world’s population and tens of millions of deaths since the first outbreak in late 2019 (1, 2). COVID-19, evoked by severe acute respiratory syndrome coronavirus 2 (SARS-CoV-2) (3), represents a highly contagious respiratory disease and has been proclaimed a global health issue by the World Health Organization. Hematologic factors including lymphopenia and prolonged prothrombin time have been presented besides a wide range of influenza- and cold-associated symptoms. Meanwhile, COVID-19 patients may have skin involvement manifested with maculopapular rash, papules, urticaria, painful acral reddish-purple papules, livedo reticularis, nonspecific lesions, and petechiae (4, 5).

Although vaccination and infection control measures worldwide have yielded certain results (6), our understanding of the spread and impacts of COVID-19 remains limited. In response to the COVID-19 pandemic, an in-depth understanding of its risk factors and associated influencing factors is quite necessary (7).

Herpes viridae represents a large family of DNA viruses, which contains 8 human-associated viruses (8). Two of the most common viruses are herpes simplex virus type-1 (HSV-1) and type 2 (HSV-2) (9). Infections caused by these two viruses are widespread worldwide and are potentially chronic in nature. HSV-1 is primarily transmitted through saliva and direct contact, usually manifested with herpes around the lips, mouth, and face accompanied by pain and itching (10). In addition to oral infections, HSV-1 leads to genital herpes (11). HSV-1 infection usually occurs during childhood or adolescence, but this virus has a lifelong latent ability and causes re-infection at any time (12). HSV-2 is transmitted dominantly *via* sexual contact and is usually characterized by herpes and ulcers in the genital area, accompanied by pain, burning sensation, and tingling of the urethra (13). HSV-2 infection generally occurs among sexually active adults, especially in populations at high risk for sexually transmitted diseases (14). Like HSV-1, HSV-2 has latent ability (15); once infected, it establishes life-long latent infection and reactivates under specific conditions (16).

Several observational studies have stated that COVID-19 infection is relevant to an elevated risk of HSV infection. For instance, Haesong et al. reported a case of herpetic gingivostomatitis two weeks after COVID-19 infection (17), and herpes symptoms were attenuated following valaciclovir treatment. Another study reported that herpes zoster virus was reactivated in some patients after COVID-19 vaccination (18). To more accurately evaluate the causal link between COVID-19 infection and HSV infection, Mendelian randomization (MR) approaches will be applied in this study.

MR methods can mimic natural random assignment conditions with genetic variants as instrumental variables, thereby addressing the frequently raised issues of reverse causation and confounding factors in observational studies (19). MR methods have higher internal validity and less risk of bias than traditional observational studies (20). We will perform MR analysis using the latest public health data and Genome-Wide Association Study (GWAS) data to validate the causal relationship between COVID-19 infection and HSV infection with COVID-19 infection as the exposure and HSV infection as the outcome. Our purpose was to determine and discuss whether COVID-19 infection was causally related to HSV infection in detail.

In the past few years, MR methods have been widely used in epidemiological studies and have yielded important results in many research fields. For example, MR methods were applied to identify the causal relation between HDL-C and coronary heart disease (CHD) (21). By analyzing large-scale GWAS data and clinical data, the researchers found a protective role of HDL-C levels against the risk of CHD. These data provided strong evidence for our knowledge of the causality between HDL-C and CHD and also demonstrated the potential of MR methods to address the issue of causality.

Based on previous research results and the successful application of MR methods, we will use the latest public health data and GWAS data and conduct MR analysis with COVID-19 infection serving as the exposure and HSV infection as the outcome for verifying the causality between them. We will discuss their causal relationship and further investigate the impacts of this relationship on the severity of COVID-19 infection and the risk of hospitalization. Through this study, we would like to offer a more accurate scientific basis for the prevention and control of COVID-19 and provide strong support for the development of relevant public health policies and interventions.

This study was expected to give a more accurate scientific foundation for the prevention and control of COVID-19 as well as powerful support for developing relevant public health policies and interventions. Altogether, the correlation between COVID-19 infection and HSV infection has been a research field that attracted great attention. Using MR approaches, we will delve into the causal link between these two and further understand the impacts of this relationship on COVID-19 infection. By filling the gaps in existing studies, we expect to offer a deeper insight into the prevention and control of COVID-19, contributing to the improvement of global public health.

## 2 Materials and methods

### 2.1 Selection of data sources and IVs for HPV infection

#### 2.1.1 Study design

**Figure 1:**
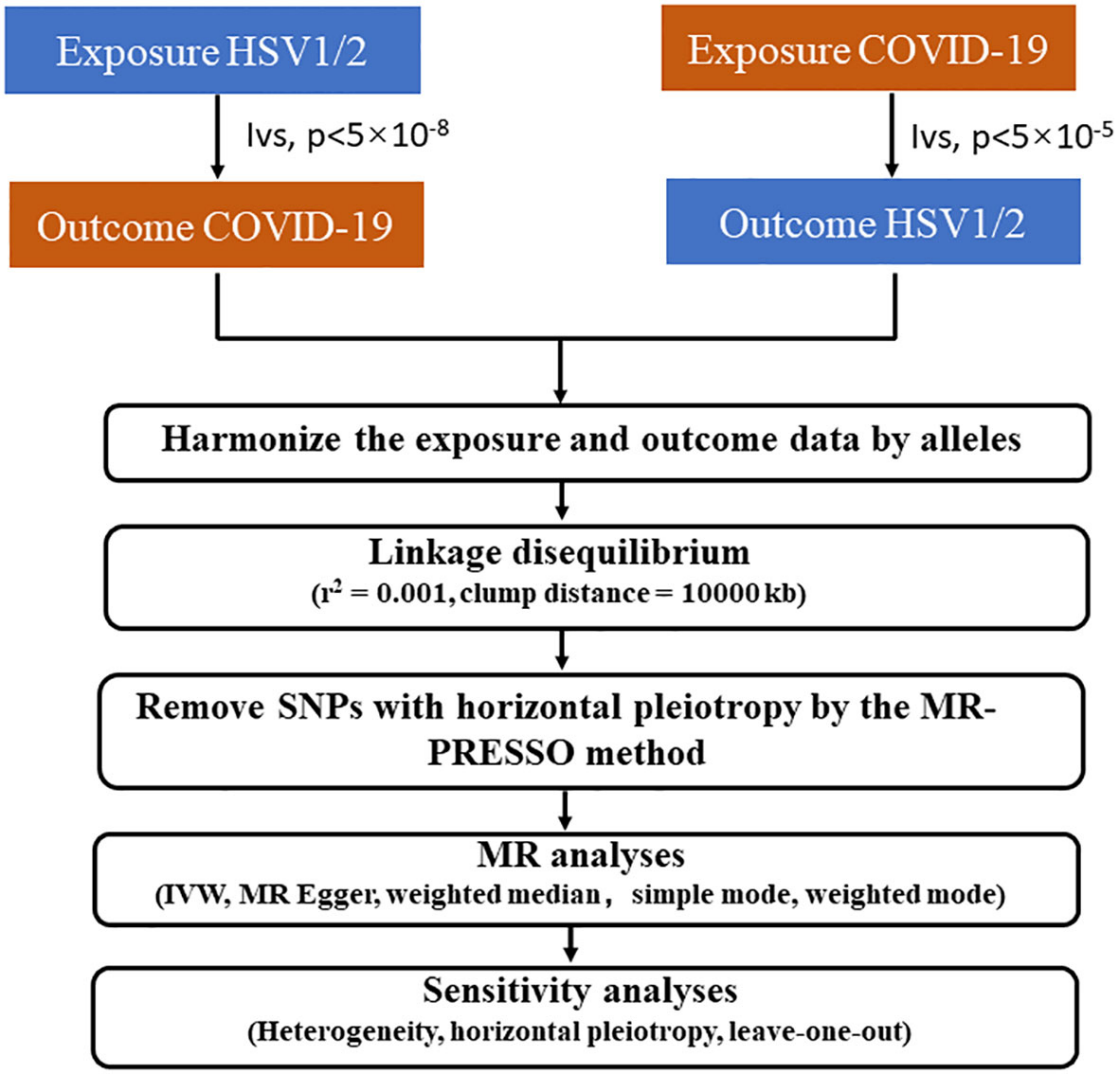
The flow diagram of the Mendelian randomization (MR) study.

This MR study conformed to the STROBE-MR guidelines (22). The study met the following 3 essential conditions: (1) IV is strongly linked to exposure; (2) IV was not associated with confounding factors; (3) IV can only affect outcomes through exposure without the involvement of other pathways. **Figure 1** depicts the study design using bidirectional MR analysis to examine the causal relationship between HSV1/2 and COVID-19. Firstly, COVID-19 was selected as the exposure and categorized into three types (severe COVID-19, hospitalized COVID-19, and SARS-CoV-2 infection) depending on the degree of COVID-19 exposure, and HSV1/2 was chosen as the outcome, through which we analyzed whether could COVID-19 affect the onset of HSV1/2; Next, with HSV1/2 serving as an exposure, we explored whether HSV infection expedited the onset of COVID-19 infection. SNPs linked to COVID-19 were screened using p < 5 × 10^-8^; to eliminate the influence of linkage disequilibrium (LD), a clumping method (r^2^ < 0.001, clumping distance = 10,000 kb) was utilized, by which we can confirm no LD between SNPs; If the minor allele frequency (MAF) <0.01, the corresponding SNPs were excluded. Lastly, HSV infection-associated SNPs were inquired using Phoenscanner and excluded. The intensity of IVs as calculated: F = (N - k - 1)/k × R^2^/(1 - R^2^), wherein N denotes the numberof samples exposed to the GWAS research, k denotes the number of IVs, and R^2^ refers to the proportion of the variance in the exposure variable explained by the IVs. Weak IV was interpreted with F<10.

#### 2.1.2 Data sources

##### 2.1.2.1 GWAS on COVID-19

COVID-19-associated data were downloaded from the COVID-19 Host Genetics Initiative GWAS (7th Edition) (https://www.covid19hg.org/results/r7/). The following 3 categories of data were included: COVID-19 infection (total cases = 13769, total controls = 1072442), COVID-19 hospitalization (total cases = 32519, total controls = 2062805), and severe COVID-19 (total cases = 122616, total controls = 2475240).

##### 2.1.2.2 GWAS on HSV

Genetic associations for HSV infections were gained from the study of Guillaume et al. who performed serologic measurements of 20 different microorganisms on 8735 European individuals using the Luminex 100 platform. Pathogens with >15% seropositivity were selected for the GWAS, which included a positive rate of 69% for HSV1 and a positive rate of 15.4% for HSV2.

#### 2.1.3 Statistical analysis

The causal correlation of COVID-19 infection with HSV infection was analyzed with the application of MR-Egger, weighted median, simple mode, weighted mode, and inverse variance weighted (IVW) methods. IVW, one of the most frequently utilized methods in MR analyses, refers to the inverse variance weighted according to the variance of each IV, and the estimates of different IVs are weighted and averaged, yielding the eventual causal estimates, which can ensure the robustness and reliability of the results (23). The MR-Egger test and the MR-PRESSO global test were used for identifying horizontal pleiotropy (24). The outliers among SNPs were examined by the MR-PRESSO test (25). With the removal of outliers, MR-Egger and MR-PRESSO tests were carried out until no SNP with horizontal pleiotropy existed in all IVs. Leave-one-out analyses were adopted for assessing the possible influence of individual SNPs on MR effects to ensure that the gained causal effect estimates were reliable and stable (26). Heterogeneity between IVs was quantified with the assistance of Cochran’s Q statistic. In this study, three categories of COVID-19 infections were analyzed, and the assessment for the causal link between COVID-19 and HSV was realized with a bidirectional, two-sample MR research. Two software packages “TwoSampleMR” and “MRPRESSO” in R version 4.1.2 were adopted for whole data processing.

## 3 Results

### 3.1 Causal effects of COVID-19 infection on HSV1 infection

**Figure 2:**
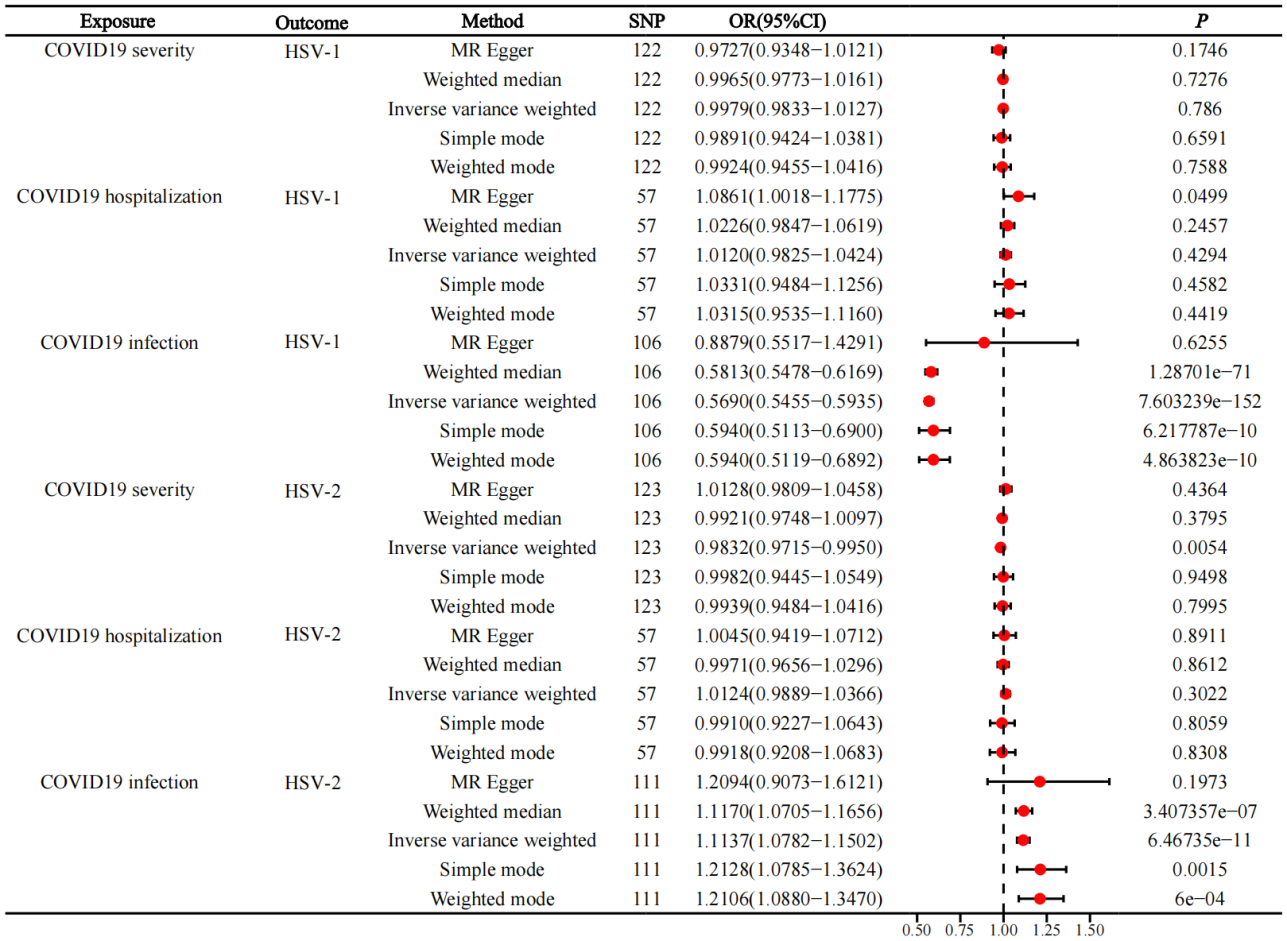
Forward MR, forest plot of MR analysis between COVID-19 infection and HSV infection.

Herein, 106, 57, and 122 SNPs were employed to identify causal correlations of HSV1 infection with COVID-19 infection, COVID-19 hospitalization, and severe COVID-19, respectively. IVW analysis showed an association between genetically predicted COVID-19 infection and reduced risk of HSV-1 infection (p=7.603239e−152, OR=0.5690, 95%CI=0.5455−0.5935). Additionally, the results of weighted median (p=1.28701e−71, OR=0.5813, 95%CI=0.5478−0.6169), simple mode (p=6.217787e−10, OR=0.5940, 95%CI=0.5113−0.6900), and weighted mode (p=4.863823e−10, OR=0.5940, 95%CI=0.5119−0.6892) methods also exhibited consistent tendency. MR-Egger did not show a statistical difference but still suggested the same detection tendency as the aforementioned four methods. COVID-19 hospitalization and severe COVID-19 were not causally linked to HSV1 infection. The results are summarized in **Figure 2**.

### 3.2 Causal impacts of COVID-19 infection on HSV2 infection

Among the three COVID-19 exposures, only COVID-19 infection was revealed to be associated with HSV-2 infection, while COVID-19 hospitalization and severe COVID-19 were not related to HSV-2 infection. The IVW results displayed that COVID-19 infection was related to a raised risk of HSV-2 infection (p=6.46735e−11, OR=1.1137, 95%CI=1.0782−1.1502). Meanwhile, weighted median (p=3.407357e−07, OR=1.1170, 95%CI=1.0705−1.1656), simple mode (p=0.0015, OR=1.2128, 95%CI=1.0785−1.3624), and weighted mode (p= 6e−04, OR=1.2106, 95%CI=1.0880−1.3470) also yielded same tendency. Although MR-Egger did not show a statistical difference, it exhibited the same detection tendency as the other four methods. The results are depicted in **Figure 2**.

### 3.3 Causal effects of HSV infection on COVID-19 infection

**Figure 3:**
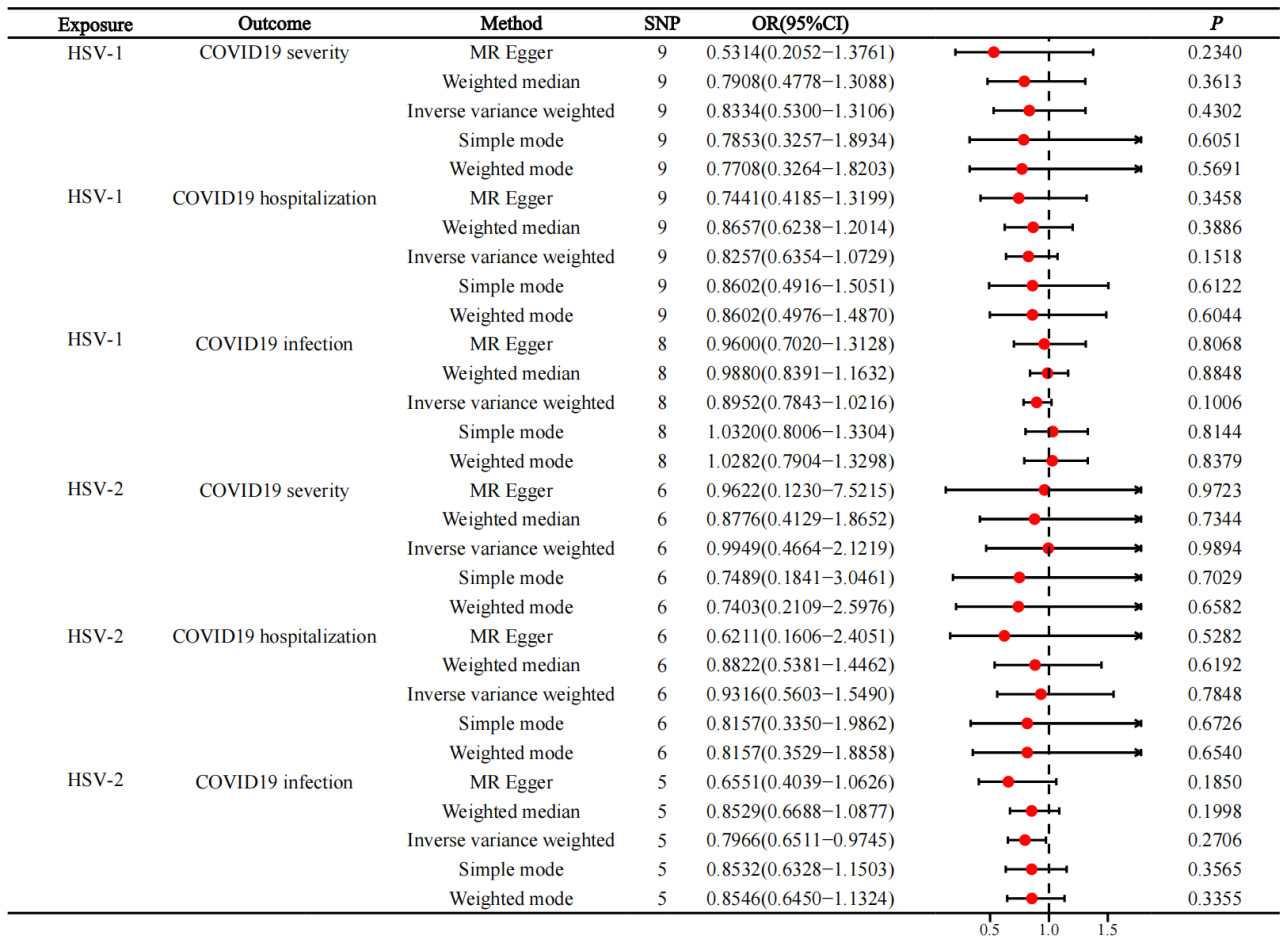
Reverse MR, forest plot of MR analysis between HSV infection and COVID-19 infection.

Additionally, HSV1 infection was not causally related to severe COVID-19 (OR=0.8334, 95%CI=0.5300−1.3106, p=0.4302, IVW), COVID-19 hospitalization (OR=0.8257, 95%CI=0.6354−1.0729, p=0.1518, IVW), and COVID-19 infection (OR=0.8952, 95%CI=0.7843-1.0216, p=0.1006, IVW). Also, HSV2 infection was not causally related to severe COVID-19 (OR=0.9949, 95%CI=0.4664−2.1219, p=0.9894, IVW), COVID-19 hospitalization (OR=0.9316, 95%CI=0.5603−1.5490, p=0.7848, IVW), and COVID-19 infection (OR=0.7966, 95%CI=0.6511−0.9745, p=0.2706, IVW) (**Figure 3**).

### 3.4 Pleiotropy test and sensitivity assessment

No heterogeneity was noted among the chosen IVs. On the grounds of MR-Egger regression and MR-PRESSO global tests, no horizontal pleiotropy existed between IVs and outcome. The results were demonstrated to be not driven by any SNP based on the leave-one-out test data. The data from pleiotropy and sensitivity assessments are displayed in **Table 1**.

**Table 1:** Heterogeneity and pleiotropy analyses.

Exposure	Outcome	Heterogeneity			MR‐Egger regression		MR-PRESSO
		Method	Q	Q-Pvalue	Intercept	p_intercept	Global test P
COVID-19 severity	HSV1	MR-Egger	41.4122	0.9999	0.0039	0.1758	0.9999
		IVW	43.2669	0.9999			
COVID-19 hospitalization	HSV1	MR-Egger	11.5262	0.9999	-0.0064	0.0706	0.9999
		IVW	14.9241	0.9999			
COVID-19 infection	HSV1	MR-Egger	19.01223	0.9999	0.0076	0.0686	0.9999
		IVW	22.3968	0.9999			
COVID-19 severity	HSV2	MR-Egger	122.4186	0.4468	-0.0044	0.0533	0.3930
		IVW	126.2710	0.3771			
COVID-19 hospitalization	HSV2	MR-Egger	28.0925	0.9990	0.0007	0.7971	0.9200
		IVW	28.1593	0.9993			
COVID-19 infection	HSV2	MR-Egger	58.5136	0.9998	-0.0026	0.5724	0.9992
		IVW	58.8342	0.9999			

## 4 Discussion

Herein, we probed into the causality between COVID-19 exposure and the risk of HSV infection using a bidirectional, two-sample MR approach and further classified the data according to the degree of COVID-19 infection as COVID-19 infection, COVID-19 hospitalization, and severe COVID-19 (27–29); HSV infections were categorized into HSV1 infection and HSV2 infection depending on the serum exposure type IgG1/IgG2 (30). This is currently considered the first MR analysis study on the relationship between COVID-19 exposure and the risk of HSV1/2 infection.

Through our MR results, we noted that COVID-19 infection exerted different impacts on the risk of HSV1/HSV2 infection. Regarding the causal influence of COVID-19 infection on HSV1 infection, our analysis suggested that COVID-19 infection was associated with a reduced risk of HSV1 infection, which was validated using a variety of causal estimation methods, including IVW, weighted median, simple mode, and weighted mode. The same detection tendency was observed by MR-Egger analysis although no statistical difference was detected.

However, COVID-19 infection was linked to an elevated risk of HSV2 infection. Similar to the results of the HSV1 infection-related analysis, this association was confirmed by a variety of causal estimation methods (IVW, weighted median, simple mode, and weighted mode). MR-Egger test failed to show a statistical difference but demonstrated the same detection trend as the aforementioned four methods. This result suggested that COVID-19 infection might increase the risk of HSV2 infection, which warranted an in-depth exploration.

So far, COVID-19 outbreaks have been associated with increased herpes virus infections (31). However, a direct association between COVID-19 and herpes zoster has not been identified. As a result of the COVID-19 pandemic in Turkey, herpes zoster, urticaria, pityriasis rosea, and other sexually transmitted diseases significantly increased (32, 33). A study from Brazil also showed that during the COVID-19 pandemic, there was an average increase of 10.7% in herpes zoster cases per million inhabitants in all regions of Brazil during the same period in 2020 compared to the number of herpes zoster cases in 2017-2019 (34). In other words, this does not directly indicate that COVID-19 has caused an increase in herpes virus cases. Lymphopenia and reductions in CD4+ T-cells, CD8+ T-cells, B-cells, and natural killer cells were observed in more than 70% of patients with no coronary pneumonia, with depletion of CD4+ and CD8+ T-cells being the most pronounced (35). A lymphocytopenia and impaired CD4+ T cell response can affect antiviral protection. Neococcal pneumonia is often accompanied by other viral infections, including influenza virus, metapneumovirus, and HHV-6. Since cellular immunity is thought to play an important role in preventing herpes infections (36), and the risk of developing herpes virus increases when VZV-specific memory T cells fall below a threshold (37), functional impairment of T cells may predispose COVID-19 patients to herpes. Given the potential association between COVID-19 and HSV-2 activation, serum antigen levels of IgG1/IgG2 in confirmed COVID-19 patients can be detected to assess the presence or absence of co-infection with HSV virus. Also when symptoms such as herpes labialis and genital herpes are present one should be alert to the possibility of co-infection with HSV. In addition, in our study, patients with COVID-19 infection had an increased risk of HSV-2, whereas no association with the risk of HSV development was found in patients hospitalized and critically ill with COVID-19, suggesting that if HSV-2 infection occurs, the patients are mostly mildly ill with COVID-19. Considering that the COVID-19 vaccine uses a weak viral infection to enable the body to acquire antibodies, and that herpes zoster infections have been reported after COVID-19 vaccination (38, 39), it remains to be seen whether HSV infections are activated after COVID-19 vaccination.

The reason for the reduced risk of HSV1 in patients with COVID-19 infection may be related to cross-immune protection, where a specific immune response forms immune memory cells after the host is infected with COVID-19. These immune memory cells can remain in the body for a long time, and they can rapidly initiate an immune response against the pathogen once similar antigenic structures are encountered again (40). Thus, it may therefore be possible for immune memory cells to recognize and attack HSV-1 faster when a host is exposed to HSV-1, thereby reducing the likelihood of infection with HSV-1.

In addition, we analyzed the causal effect of HSV infection on COVID-19. According to our study, neither HSV1 nor HSV2 infections were causally linked to severity, hospitalization risk, or infection severity. This suggests that HSV infection may not be a major causal factor in these COVID-19-related indicators. However, to fully assess the relationship between HSV infection and COVID-19, we must consider other influencing factors.

In our study, we also analyzed pleiotropy and sensitivity and did not find any heterogeneity between the selected IVs, and the MR-Egger regression and MR-PRESSO global tests showed no horizontal pleiotropy between IV and outcomes. Leaving one out of the analysis revealed that no single SNP drove the results. Our causal estimation results are further supported by these results, which demonstrate their robustness and reliability.

## 5 Conclusions

In summary, our findings suggest that COVID-19 infection has different effects on the risk of HSV1 and HSV2 infection. COVID-19 infection may reduce the risk of HSV1 infection but increase the risk of HSV2 infection. However, there was no causal relationship between HSV infection and COVID-19 infection severity, hospitalization risk, or overall infection risk. Our study provides valuable information to further understand the complex relationship between COVID-19 infection and HSV infection and provides a scientific basis for the development of public health policies and interventions. However, we also need to be aware of the limitations of the study and encourage future studies to explore this area in depth with larger samples and more factors considered.

## Data availability statement

The original contributions presented in the study are included in the article/supplementary material. Further inquiries can be directed to the corresponding authors.

## Ethics statement

The manuscript presents research on animals that do not require ethical approval for their study.

## Author contributions

MY: Data curation, Formal analysis, Methodology, Writing – original draft, Writing – review & editing. L-YX: Formal analysis, Funding acquisition, Visualization, Writing – original draft. MG: Data curation, Funding acquisition, Project administration, Writing – review & editing. RF: Formal analysis, Funding acquisition, Methodology, Writing – review & editing. RS: Formal analysis, Methodology, Resources, Writing – review & editing. L-LF: Formal analysis, Software, Writing – original draft. H-CF: Data curation, Formal Analysis, Funding acquisition, Project administration, Writing – review & editing. SB: Formal analysis, Investigation, Writing – review & editing.
